# Simple and Rapid Method for the Determination of Uric Acid-Independent Antioxidant Capacity

**DOI:** 10.3390/molecules16087058

**Published:** 2011-08-17

**Authors:** Darko Duplancic, Lea Kukoc-Modun, Darko Modun, Njegomir Radic

**Affiliations:** 1 Department of Cardiology, University Hospital Split, Soltanska 1, School of Medicine, University of Split, Soltanska 2, 21000 Split, Croatia; Email: darko.duplancic@gmail.com (D.D.); 2 Department of Analytical Chemistry, Faculty of Chemistry and Technology, University of Split, Teslina 10/V, 21000 Split, Croatia; Email: njegomir.radic@gmail.com (N.R.); 3 Department of Pharmacology, School of Medicine, University of Split, Soltanska 2, 21000 Split, Croatia; Email: drmodun@gmail.com (D.M.)

**Keywords:** antioxidant, uric acid, FRAP, uricase

## Abstract

Determination of the relative contribution of uric acid level increases to the total measured antioxidative activity could be very useful for testing antioxidative products and their effect on human health. The aim of this report is to present a simple spectrophotometric method that combines the measurement of total antioxidative capacity of a sample by ferric reducing/antioxidative power (FRAP) assay, with the uricase-reaction (specific elimination of uric acid), in order to establish and correct for the contribution of uric acid in FRAP values. We measured FRAP values, with (uric acid-independent antioxidant capacity, TAC-UA) and without (total antioxidant capacity, TAC) uricase treatment, and expressed it as μmol/L of uric acid equivalents. In such way, it was possible to determine both total and uric acid-independent antioxidant capacity, plasma uric acid (UA, as the difference between TAC and TAC-UA), and the ratio of the uric acid in total antioxidant capacity (UA/TAC).

## 1. Introduction

Testing for antioxidant activity/capacity of new synthesized chemical compounds, nutraceuticals or extracts from natural sources, is a rational method of screening for future products with potential beneficial impact on human health [1,2]. The methods used are usually based on an antioxidative capacity, or free radical quenching activity of the sample [3,4,5]. Nevertheless, occasionally there is a discrepancy between the antioxidative activity, measured *in vitro*, and the antioxidative effects observed *in vivo* in human subjects after consumption of the product [6]. One possible deviation when a strong *in vitro* antioxidant does not show the same beneficial effect *in vivo*, is most likely due to insufficient absorption in the gastrointestinal tract. On the other hand, some chemically “redox inactive” compounds, like fructose [7] or mixtures of ethanol and glycerol [8,9], showed strong antioxidant effects *in vivo*, due to a specific metabolic effect (not possible *in vitro*)—an increase of plasma uric acid concentration. Even for the well-established antioxidants, like polyphenols [10], the effect of their consumption on human health has been questioned. In fact, it has been shown that the increase in human plasma antioxidant capacity after apple consumption is due to the metabolic effect of fructose (from apple) on plasma uric acid elevation, and not due to any apple-derived antioxidant polyphenols [7]. Our team has recently shown that increase in human plasma antioxidant capacity after red wine consumption is due to both wine polyphenols and increase of plasma uric acid [9], and that the glycerol and ethanol in red wine are responsible for uric acid-related increases in plasma antioxidant capacity [8]. Finally, it has been suggested that the large increase in plasma total antioxidant capacity observed after the consumption of polyphenol-rich foods is not caused by the polyphenols themselves, but is likely the consequence of increased uric acid levels [11].

Uric acid is a well-known antioxidant that contributes significantly to the plasma antioxidant capacity [12,13]. However, as uric acid is a molecule with both beneficial and harmful effects on human health [14], the determination of relative contribution of plasma uric acid increase to the antioxidative activity of new products could be very useful. Therefore, we developed a simple and rapid method for determination of uric acid-independent antioxidant capacity. The main principle for the proposed method is a combined measurement of total antioxidant capacity of a sample by FRAP assay, and specific depletion of the antioxidant (uric acid) in the sample. This approach was already reported for combined determination of FRAP and ascorbic acid in plasma, the so-called FRASC assay (ascorbat oxidase was used instead of uricase) [15].

## 2. Results and Discussion

**Figure 1:**
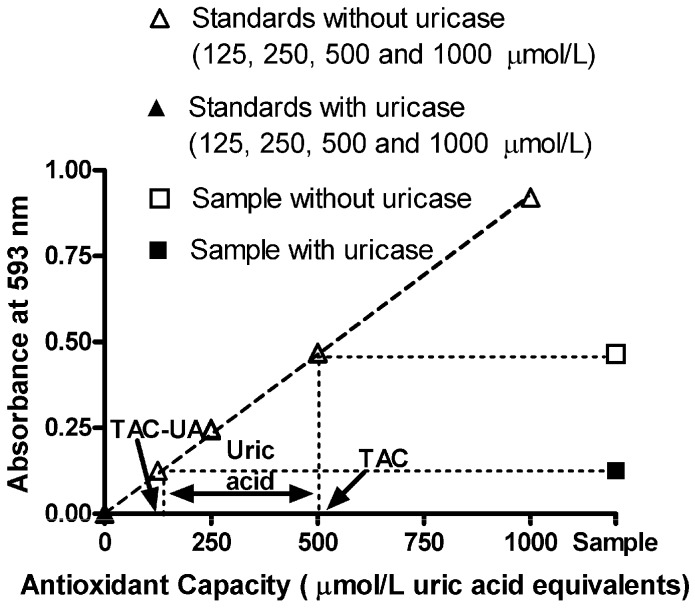
Absorbance readings *vs.* antioxidant capacity of uric acid standards (without and with uricase treatment), combined with absorbance of a sample without (total antioxidant capacity, TAC, 500 ± 6 μmol/L uric acid equivalents) and with uricase treatment (uric acid-independent antioxidant capacity, TAC-UA, 140 ± 2 μmol/L uric acid equivalents). Plasma uric acid is calculated as the difference between TAC and TAC-UA (360 ± 4 μmol/L). The ratio of the uric acid in total antioxidant capacity (UA/TAC) is 72%. All data are expressed as means ± SD. All measurements for each sample were done in triplicate.

The response in the FRAP assay with uric acid standard solutions (in the absence of uricase) was linear up to 1,000 µmol/L, and the linear regression equation was y = 0.000977x − 0.00060, r^2^ = 0.9999 (open triangles in Figure 1). Treatment with uricase completely abolished this response (closed triangles in Figure 1). Successful quantitative removal of uric acid using uricase in the proposed method was further confirmed by the reference method [16]—In uric acid standard solutions and plasma samples, there were no detectable levels of uric acid after treatment with uricase, indicating that the proposed conditions (incubation time for 20 min at 25 °C, pH 7.4) were suitable for uricase to catalyze the degradation of uric acid to allantoin and H_2_O_2_ (Table 1, 4th column).

In order to test whether uricase itself interferes with the FRAP assay, we determined TAC and TAC-U for freshly prepared standards of ascorbic acid. We found no difference between TAC and TAC-UA values for 250, 500 and 1000 µmol/L of ascorbic acid (255 ± 3, 512 ± 5 and 1016 ± 9 *vs.* 252 ± 3, 510 ± 6 and 1013 ± 8 µmol/L UA equivalents, respectively), indicating that uricase treatment (for TAC-UA samples) didn’t interfere with the FRAP assay.

**Table 1:** Original data used for testing the proposed method for determination of plasma uric acid (UA)-calculated as the difference between TAC and TAC-UA. All data are expressed as means ± SD. All measurements for each sample were done in five replicates.

Sample number	UA (μmol/L) Proposed method	UA (μmol/L) Reference method [16]		UA (μmol/L) Proposed method Sample		
		Without uricase	With uricase	+100 μmol/L UA [recovery %]	+250 μmol/L UA [recovery %]	+500 μmol/L UA [recovery %]
1	314 ± 3	321 ± 1	0	411 ± 5 [97%]	556 ± 6 [97%]	800 ± 9 [97%]
2	352 ± 4	360 ± 1	0	448 ± 5 [97%]	600 ± 7 [99%]	840 ± 10 [98%]
3	327 ± 3	326 ± 1	0	430 ± 4 [103%]	580 ± 6 [101%]	833 ± 8 [101%]
4	365 ± 4	371 ± 1	0	461 ± 5 [96%]	610 ± 7 [98%]	870 ± 9 [101%]
5	381 ± 4	385 ± 1	0	479 ± 5 [98%]	623 ± 7 [97%]	871 ± 9 [98%]

As the enzymatic reaction of uric acid with uricase produces H_2_O_2_, we tested whether the newly formed H_2_O_2_ could interfere with the other antioxidants in the sample, particularly ascorbic acid (the main contributor to FRAP values of plasma, after uric acid [12]). In order to test this we fortified the freshly prepared standards of ascorbic acid (250, 500 and 1,000 µmol/L) with 500 µmol/L of uric acid, and determined TAC and TAC-U for these samples. We found a significant difference between TAC and TAC-UA values (750 ± 6, 1,009 ± 9 and 1,511 ± 12 *vs.* 249 ± 3, 504 ± 5 and 1,002 ± 7 µmol/L UA equivalents, respectively). However, there was no significant difference between TAC-UA values for 250, 500 and 1,000 µmol/L of ascorbic acid that was exposed to H_2_O_2_ (produced after uricase reaction with uric acid) in comparison to the “unexposed” one (no uric acid present, therefore no H_2_O_2_ produced), 249 ± 3, 504 ± 5 and 1,002 ± 7 µmol/L *vs.* 252 ± 3, 510 ± 6 and 1,013 ± 8 µmol/L UA equivalents, respectively, indicating that the newly formed H_2_O_2_ didn’t interfere with the other antioxidants (ascorbic acid) in the sample. The proposed method was then tested for the determination of plasma uric acid (UA)-calculated as the difference between TAC and TAC-UA. The original data used for testing the proposed method for determination of UA is presented in Table 1.

When we used Bland-Altman analysis (plot of differences *vs.* means of results by two methods) to compare the results of plasma uric acid obtained by proposed method with the automated uricase method [16], we found acceptable bias ±95% limits of agreement (−5 ± 7 µmol/L) of uric acid values. The analytical recovery of uric acid (concentration ranging from 100 to 500 µmol/L) added to plasma samples was 98 ± 3%. The coefficient of variation of all determination by the proposed method was 3%.

After this testing of the method, we determined TAC, TAC-UA and UA in our plasma samples, as illustrated in Figure 1. The original data of TAC, TAC-UA and UA in our plasma samples is presented in Table 2. Values of total antioxidant capacity (TAC), uric acid-independent antioxidant capacity (TAC-UA) and uric acid (UA) for our samples were 518 ± 25 and 175 ± 12 μmol/L uric acid equivalents and 345 ± 15 μmol/L respectively (means ± SEM). The values of plasma uric acid, obtained by the proposed method (Table 2, 4th Column) are comparable with those obtained by the reference method [16] (Table 2, 5th Column), as indicated by statistical analysis (P = 0.88, Student *t*-test). On the other hand, the values of TAC are significantly higher in comparison to TAC-UA (P < 0.0001, Student *t*-test), indicating that uric acid is the main contributor to the total antioxidant capacity in human plasma, when measured by the FRAP method. Indeed, the calculated ratio of the uric acid in total antioxidant capacity (UA/TAC) was relatively constant at 67%, with small SD (3%). These findings are in accordance with previous studies that showed that uric acid is contributing to approximately 70% of plasma total antioxidant capacity, when measured by FRAP method [9,12].

**Table 2:** Original data of TAC, TAC-UA and UA in our plasma samples. All data are expressed as means ± SD. All measurements for each sample were done in triplicate.

Sample number	TAC (μmol/L UA equivalents)	TAC-UA (μmol/L UA equivalents)	UA (μmol/L)	
			Proposed method	Reference method [16]
1	488 ± 4	174 ± 2	314 ± 3	321 ± 1
2	535 ± 5	183 ± 2	352 ± 4	360 ± 1
3	515 ± 5	188 ± 2	327 ± 3	326 ± 1
4	544 ± 6	179 ± 3	365 ± 4	371 ± 1
5	551 ± 5	170 ± 2	381 ± 4	385 ± 1
6	399 ± 4	144 ± 2	255 ± 3	260 ± 1
7	500 ± 6	140 ± 2	360 ± 4	361 ± 1
8	614 ± 7	223 ± 3	391 ± 4	395 ± 1
9	397 ± 4	109 ± 2	288 ± 3	292 ± 1
10	644 ± 7	229 ± 3	415 ± 5	411 ± 1

**Figure 2:**
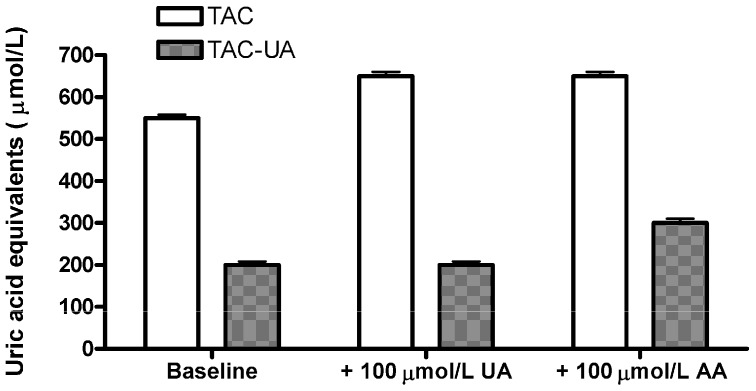
Determination of total and uric acid-independent antioxidant capacity (TAC and TAC-UA, respectively) for a regular sample (baseline), the sample with extra 100 μmol/L of uric acid (UA) and the sample with extra 100 μmol/L of ascorbic acid (AA). All data are expressed as means ± SD. All measurements for each sample were done in triplicate.

In order to confirm that the proposed method specifically eliminates the relative contribution of uric acid in total antioxidant capacity of plasma samples, we have added 100 μmol/L of uric acid (UA) or 100 μmol/L ascorbic acid (AA) to a plasma sample (Figure 2). The ascorbic acid was selected as it is a strong antioxidant which shares a 1:1 stoichiometric equivalence with the uric acid in the FRAP assay [12]. Addition of uric acid resulted in an elevation of TAC (from 550 ± 6 to 648 ± 8 µmol/L), but the TAC-UA remained virtually unchanged (from 201 ± 3 to 205 ± 4 µmol/L), while addition of ascorbic acid resulted in an elevation of both TAC (from 550 ± 6 to 645 ± 9 µmol/L), and TAC-UA (from 201 ± 3 to 295 ± 6 µmol/L). This result confirmed that the proposed method is selective for depletion of relative contribution of uric acid in total antioxidant capacity, as there was not any significant increase of TAC-UA after addition of UA. Moreover, as the measured increase of TAC-UA after addition of AA (94 ± 6 µmol/L uric acid equivalents) is in agreement with the given amount of AA (100 µmol/L) it is likely that relative contribution of other antioxidants to total antioxidant capacity of the sample will not be underestimated by the proposed method, as they are not affected by the uricase reaction. As ascorbic acid is very sensitive to oxidation, we used only freshly prepared standards, and fresh samples (heparinized plasma), in accordance to a previous report [17].

**Figure 3:**
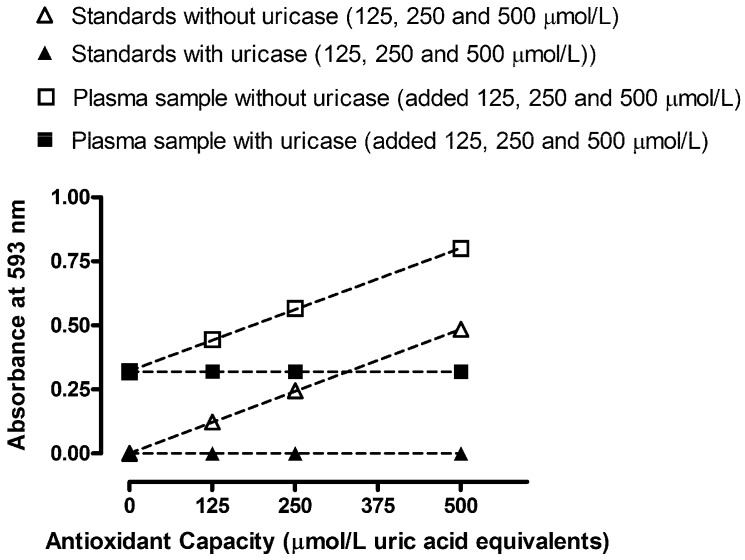
Absorbance readings *vs.* antioxidant capacity of uric acid standards (without and with uricase treatment) combined with standard addition experiment performed on human plasma (without and with uricase treatment). All data are expressed as means ± SD. All measurements for each sample were done in triplicates.

In order to observe any interfering effects of plasma constituents for determination of TAC and TAC-UA, we performed standard addition experiments of uric acid (before and after the uricase reaction) on human plasma, as shown in Figure 3. The obtained response-concentration lines are virtually parallel in the standard addition tests (slope values were 0.00097 *vs.* 0.00096 for standards and plasma samples without uricase, respectively). Therefore, we can conclude that plasma constituents did not cause any detectable chemical deviation from Beer’s law.

We performed additional testing in order to establish whether hyperglycemia or hyperlipemia could influence the determination of TAC and TAC-UA. We mixed a fresh plasma sample with increased triglyceride and TAC levels, proportionally with a sample with low triglyceride and TAC levels. Triglyceride concentrations up to 14 mmol/L had no effect on recovery. In a second experiment we mixed a fresh plasma sample with increased glucose and TAC levels, proportionally with a sample with low glucose and TAC levels. Glucose concentrations up to 18 mmol/L had no effect on recovery. Finally, we tested the same blood sample in four different vacutainers in order to establish whether any common anticoagulant (EDTA, heparin, citrate and fluoride oxalate) could influence the determination of TAC and TAC-UA. We found a significant reduction in both TAC (>5%) and TAC-UA (>30%) in the EDTA plasma. The reduction of TCA and TAC-UA values in EDTA plasma could be due to the increased loss of ascorbic acid in the EDTA plasma [17]. However, there is another possible explanation. The FRAP reagent—as well as other transition metal ion-based reagents—is expected to be strongly affected by EDTA, preferentially chelating the higher oxidation state of iron (*i.e.*, Fe(III)) compared to the lower oxidation state [18]. Thus, Fe(III)/Fe(II) reduction potential would decrease in the presence of EDTA, making Fe(III)-TPTZ a weaker oxidant, incapable of oxidizing all antioxidant constituents of serum. Consequently, the measured TAC values would be lower in the presence of EDTA. Therefore, we can conclude that EDTA is not an appropriate anticoagulant for determination of TAC and TAC-UA.

The approach of separating the role of uric acid (or ascorbic acid) is not new, and it has been proposed in previous studies. Benzie *et al*. proposed the specific elimination of ascorbic acid impact in the FRAP assay (so-called FRASC assay), which is very similar to the proposed method [15,19]. Different studies have emphasized the importance, or suggested an actual method of determination, of total antioxidant capacity without the effect of uric acid in different samples [7,9,13,15,20,21]. This report follows the suggestions and ideas derived from the abovementioned studies. However, we think that the proposed method represents a major improvement of the original FRAP assay, as the FRAP assay is strongly influenced by uric acid (60%–70%). Benzie and Strain, the creators of the original FRAP assay [12] and the modified “FRASC” assay [15], have indirectly acknowledged our opinion: “The non-UA FRAP is a novel and potentially useful index of antioxidant defense. While uric acid appears to contribute around 60% of the total reducing (“antioxidant”) power of plasma, its role as a physiological antioxidant is doubtful. High total “antioxidant” status associated with elevated plasma uric acid concentrations may be misleading, masking a relative or absolute deficiency of other antioxidants” [15]. Moreover, Benzie and Strain have highlighted that: “…Subtracting this (antioxidant power of the sample due to uric acid) from the FRAP value of the sample gives the nonuric acid FRAP value, which may offer a more sensitive index of antioxidant status in uric acid-rich fluid such as plasma” [19]. The possibility of using the proposed method for determination of TAC and TAC-UA in other biological samples that are often used like tears [22] or urine [21], represents a new challenge for the future.

Finally, the measurement of total antioxidant capacity is rather a “concept” than an actual analytical measurement of a specific analyte. An increased antioxidant capacity in plasma may not necessarily be a desirable condition if it reflects a response to increased oxidative stress. Similarly, a decrease in plasma or serum antioxidant capacity may not necessarily be an undesirable condition if the measurement reflects decreased production of reactive species. Therefore, no single measurement of antioxidant status is going to be sufficient. Antioxidant capacity could be useful as a simple initial test for diagnosis of oxidative stress and monitoring therapy for first orientation, to be followed by more specific analyses of individual antioxidants, using more expensive and selective equipment (like HPLC or GC-MS). Every antioxidant capacity assay has its own limitations, for instance the FRAP assay works at a non-physiological pH (3.6). Some other popular assays for total antioxidant capacity work at physiological pH (7–7.4), like CUPRAC [23] and different versions of TEAC assay [24,25]. In a clinical study it is recommended to use a “battery” of measurements, including different assays for total antioxidant capacity and different assays for oxidative stress markers (e.g., end products of lipid peroxidation) [3,4,5].

## 3. Experimental

### 3.1. General

Trolox^®^, 2,4,6-tri(2-pyridyl)-1,3,5-triazine (TPTZ), ascorbic acid, uric acid, ferric chloride hexahydrate, hydrochloric acid, sodium acetate trihydrate, uricase (lyophilized, Sigma U0880, EC number 1.7.3.3) and glacial acetic acid were purchased from Sigma-Aldrich Chemie (Taufkirchen, Germany). All solutions and reagents were made with use of deionized (Milli Q) water. Vacutainers with heparin were provided by Becton Dickinson (UK).

Absorbance measurments were performed on a Shimadzu UV-1601 UV-Vis spectrophotometer (Shimadzu, Kyoto, Japan). The “reference” method for uric acid determination, the automated uricase method [16], was performed by an Olympus AU 2700 (Olympus Michima Co.LTD, Shizouka, Japan)automatic analyzer using an enzymatic laboratory kit (Olympus, OSR 6136). This method is used routinely for both serum and plasma samples, in the Department of Laboratory Diagnostic, University Hospital Split.

The study was conducted in accordance with the Declaration of Helsinki and approved by the Ethics Committee of the University of Split School of Medicine. All subjects gave written consent prior to their participation in the study. Ten healthy male volunteers were recruited for the study. Blood samples were collected into heparin vacutainers and plasma TAC and TAC-UA values were analyzed immediately after sampling. Plasma uric acid was calculated as the difference between TAC and TAC-UA.

### 3.2. Preparation of 5 mmol/L stock Uric Acid Standard Solution and Working Solutions [16]

Dissolve uric acid (84 mg) and lithium carbonate (60 mg) in deionized water (30 mL) at 60 °C and dilute to 100 mL with deionized water. Keep refrigerated and protected from light [16]. Working uric acid standards were prepared by diluting the stock uric acid standard solution daily with deionized water, in order to make working uric acid standard solutions (500 or 1,000 μmol/L).

### 3.3. Preparation and Use of FRAP Reagent

FRAP reagent was freshly prepared by mixing (ratio 10:1:1) acetate buffer (pH 3.6, 300 mmol/L), TPTZ (10 mmol/L) in HCl (40 mmol/L) and FeCl_3_ (20 mmol/L). Plasma samples or uric acid standards (200 mL) were mixed with PBS (pH 7.4, 60 mL, for TAC) or PBS-containing uricase (10 U/mL, 60 mL, for TAC-UA) and incubated for 20 min at 25 °C. To FRAP reagent (2.25 mL) sample (75 mL) was added and, after 8 min at room temperature, absorbance at 593 nm was read *vs.* reagent blank [12,19]. The change in absorbance (D A_593nm_) was calculated for each sample and related to D A_593nm_ of uric acid standard solutions tested in parallel. All measurements were done in triplicate, except testing measurements (Table 1) that were done in five replicates 

## 4. Conclusions

The proposed method (modified FRAP assay) for determination of uric acid-independent antioxidant capacity (TAC-UA) together with total antioxidant capacity (TAC) is simple, robust and rapid. We believe it will be useful for testing new antioxidative products (and relative contribution of uric acid in the induced antioxidative effect) and their effect on human health. Moreover, the method could be used for clinical studies that are focused on antioxidant defense and oxidative stress in patients. Although the uric acid is a major determinant of antioxidant capacity, it was disputed that the observed high UA level could be a causal, compensatory, or coincidental risk factor in patients suffering from cardiovascular diseases. Therefore, as the function of uric acid in human organism is still unclear, it may be important to estimate plasma antioxidant capacity both with an without uric acid, especially in diseases that combine oxidative stress and hyperuricemia, like cardiovascular and renal diseases, gout, obesity and diabetes mellitus type 2 [5,11,13,14,26]. Future clinical studies are warranted in order to establish a clinical value of the TAC-UA, as an index of antioxidant defense.
